# Pelvic ring fracture and erectile dysfunction (PERFECD) – 3 year follow-up cross sectional study

**DOI:** 10.1007/s00068-024-02761-y

**Published:** 2025-01-28

**Authors:** Gioia Rizzoli, Florian A. Schmid, Franziska Kessler, Yannik Kalbas, Felix Karl-Ludwig Klingebiel, Till Berk, Roman Pfeifer, Daniel Eberli, Hans-Christoph Pape, Sascha Halvachizadeh

**Affiliations:** 1 Faculty of Medicine, University of Zurich, Raemistrasse 71, 8006 Zurich, Switzerland; 2 Department of Trauma, University Hospital Zurich, Raemistrasse 100, 8091 Zurich, Switzerland; 3 Department of Urology, University Hospital Zurich, Raemistrasse 100, 8091 Zurich, Switzerland; 4 Department of Trauma and Reconstructive Surgery, University Hospital RWTH Aachen, Aachen, Germany

**Keywords:** Pelvic ring fracture, Erectile dysfunction, Quality of life, IIEF-5, Trauma, Outcome

## Abstract

**Introduction:**

Pelvic ring fractures are known to be associated with complications associated with adjacent organ injuries, such as the urogenital tract (e.g. erectile dysfunction (ED), which are sometimes diagnosed in a delayed fashion. Therefore, we assessed the quality of life (QoL) and the rate of erectile dysfunction (ED) following pelvic ring fractures at a minimum of 3 years after pelvic ring injury.

**Methods:**

Between January 1, 2016, and December 31, 2020, adult male patients (≥ 18 years) with pelvic ring injuries were included in the study. Fractures were classified according to the Young & Burgess (Y&B) classification system, while pelvic contusions were categorized as the control group. Data were collected using a written questionnaire that assessed Quality of Life (QoL) by Short Form 12 (SF-12) and erectile dysfunction (ED) with the International Index of Erectile Function 5 (IIEF-5). ED was stratified as follows: no ED (21–25 points), mild ED (16–21 points), moderate ED (9–15 points), and severe ED (5–7 points). Comorbidities and risk factors for ED were also assessed, including vasculopathy, peripheral artery disease, hypercholesterolemia, coronary artery disease, diabetes, and smoking.

**Results:**

A total of 182 patients were included, with a mean age at injury of 53.5 years (SD 17.1) and a mean age at the time of the questionnaire of 57.8 years (SD 17.4). The distribution of patients was as follows: APC Group (n = 20, 11.1%), LC Group (n = 94, 52.2%), CMVS Group (n = 6, 3.3%), and Control Group (n = 60, 33.3%). The mean Injury Severity Score (ISS) was 24.6 points (SD 16.4). Regarding erectile dysfunction, 8 patients (17.4%) had no ED, 10 (21.7%) had mild ED, 6 (13.0%) had moderate ED, and 22 (47.8%) had severe ED. Quality of Life (QoL) was significantly reduced in patients with CMVS pelvic fractures, particularly in physical role function, which scored 62.5 points (SD 29.6, p < 0.001). All patients in the APC Group reported at least a mild form of ED. APC injuries were identified as an independent risk factor for lower IIEF-5 scores (OR -4.5, 95% CI -8.3 to -0.7, p = 0.02), comparable to other risk factors such as hypertension (OR -9.2, 95% CI -12.8 to -5.6, p < 0.001), diabetes (OR -5.3, 95% CI -9.4 to -1.2, p = 0.012), and smoking (OR -2.6, 95% CI -5.2 to -0.04, p = 0.05).

**Conclusion:**

Vertical shear fractures are associated with significantly lower quality of life compared to APC or LC fractures three years post-injury. The APC type of pelvic ring injury was identified as an independent risk factor for the development of erectile dysfunction (ED). Early screening and appropriate management should be initiated for patients with APC injuries to address and mitigate the risk of ED.

## Introduction

Fractures of the pelvic ring present a significant challenge for orthopedic trauma surgeons due to their complexity and their high-energy trauma [1], associated with major hemorrhage. They often necessitate immediate surgical stabilization, temporary fixation, and specific hemorrhage control techniques [2]. Additionally, pelvic ring fractures frequently lead to regional injuries, including vascular damage to the presacral venous plexus [3], urologic complications such as bladder rupture [4], or urethral injuries [5], neuro-urological issues [6], and, rarely, genital injuries [7].

In the long-term, the presence of neurologic injuries has been found to have a more substantial impact on functional outcomes compared to the overall severity of the injury [8]. Factors such as persistent pain, return to previous employment, functional scoring, and general health outcomes appear to be independent of the chosen treatment strategy [9]. The classification of the pelvic ring injury may also be linked to impaired quality of life [10].

Most outcome studies following pelvic ring injuries have predominantly focused on functional outcomes, such as the Majeed Score or Timed Up and Go tests, as well as quality of life (QoL), while less attention has been given to erectile dysfunction (ED) [11]. Numerous studies have identified medical risk factors for the development of ED, with the most commonly reported including hypertension, cardiovascular disorders, smoking, obesity, dyslipidemia, diabetes, metabolic syndrome, anxiety, and depression [12].

We hypothesize that pelvic ring fractures are not associated with the development of ED. The primary aim of this study is to assess the QoL following pelvic ring injuries and to investigate the association between these injuries and the development of ED three years post-trauma.

## Methods

The study protocol for this cross-sectional survey was approved by the cantonal ethics committee (BASEC Nr.: 2020–02811). Reporting of the results follows the consensus-based checklist for reporting of survey studies [13] and the Strengthening the Reporting of Observational Studies in Epidemiology (STROBE) statement [14].

### Patient recruitment and consent

Patients were contacted with a letter that included the standardized questionnaire assessing Quality of Life) with Short Form-12 (SF-12) Version 2.0 [15] and the International Index of Erectile Function (IIEF-5) for the assessment of Erectile Dysfunction (ED) [16]. Completion and return of the questionnaire indicated consent for the publication of anonymized data.

### Inclusion and exclusion criteria

Eligible participants were male patients, aged 18 and above, who were admitted with pelvic ring injuries between January 1, 2016, and December 31, 2020, at our trauma center. Patients were identified using the International Classification of Diseases 10 (ICD-10) Code S3, which encompasses pelvic injuries. Patients who did not return the completed questionnaire or had fractures limited to the acetabulum were excluded.

### Study groups

Pelvic ring injuries were classified according to the Young and Burgess classification [17]. This classification yielded Group APC (Anterior–Posterior Compression), Group LC (Lateral Compression), and Group CMVS (Combined Mechanism and Vertical Shear). The APC type of injury was further classified as Type 1 (less than 2.5 cm symphyseal diastasis), Type 2 (more than 2.5 cm symphyseal diastasis and disruption of the anterior SI ligaments and pubic symphysis) and Type 3 (complete disruption of both anterior and posterior SI ligaments) [17]. Patients diagnosed with pelvic soft tissue injuries in the absence of fractures or ligamentous injuries, served as the control group (Group Control).

### Variables, definitions and sample size

The primary outcome of this study was ED, assessed by the IIEF-5 questionnaire. QoL was assessed using the SF-12 questionnaire, Version 2.0. Additional variables included risk factors for ED: vasculopathy, peripheral artery disease (PAD), hypercholesterinemia requiring regular medication, coronary artery disease, hypertension requiring regular medication, diabetes, and smoking habits [12]. These variables were collected through the questionnaire and electronic medical records. The treatment of pelvic ring injuries followed local and international guidelines [1, 2]. The study aimed to include the maximum number of eligible patients to minimize selection bias. No formal sample size calculation was performed due to the study’s design.

### Statistical methods

Continuous variables are presented as mean and standard deviation (SD), and categorical variables as count (n) and percentage. The IIEF-5 scores were categorized as follows: No ED (25–21 points), mild ED (16–21 points), moderate ED (9–15 points), and severe ED (5–7 points) [11]. Group comparisons for continuous variables were performed using univariate regression analysis. Multivariate regression analyses included variables considered risk factors for ED development, with the IIEF-5 score as the outcome measure. Results are presented with Odds Ratios (OR) and 95% confidence intervals (CI). QoL and SF-12 measures were categorized according to the questionnaire guidelines, resulting in scores ranging from zero (worst outcome) to 100 (best possible outcome). All statistical analyses were performed using R (R Core Team (2023). R: A Language and Environment for Statistical Computing. R Foundation for Statistical Computing, Vienna, Austria. https://www.R-project.org/).

## Results

**Fig. 1:**
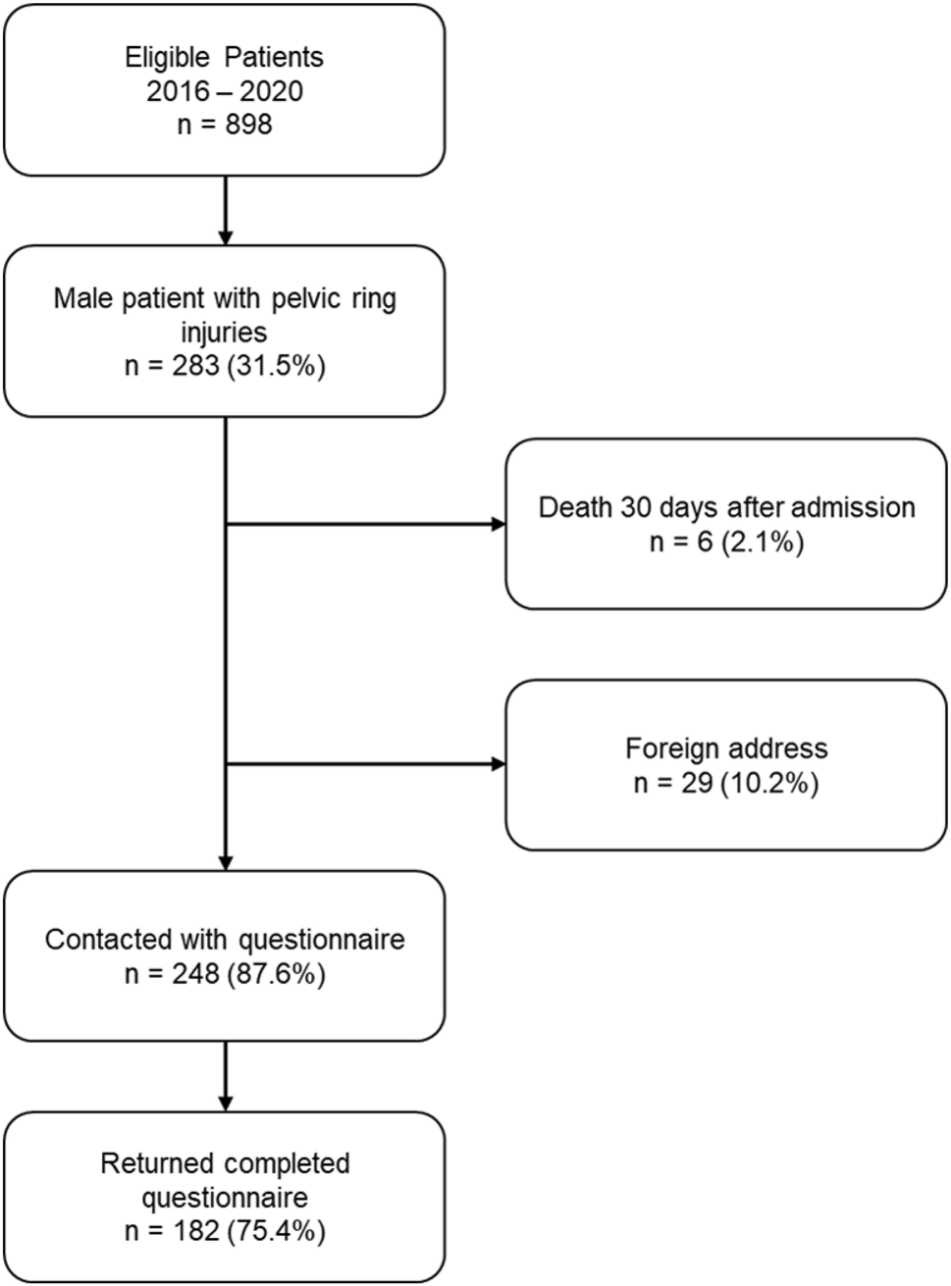
Flow Chart of the selection of the study population

**Fig. 2:**
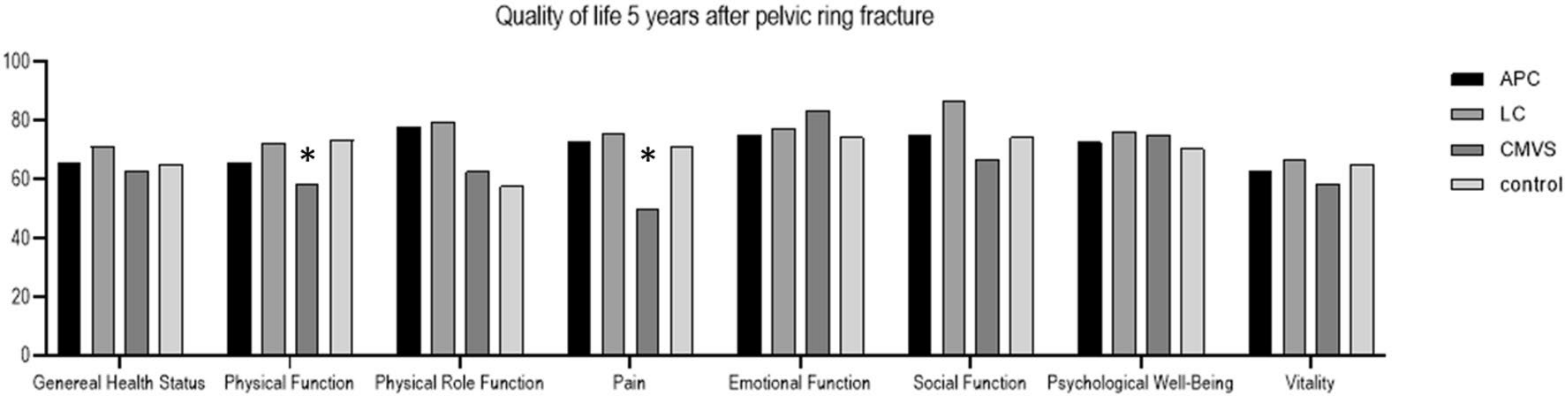
Results of the Qulality of Life questionnaire (SF-12), Group CMVS showed significant wore pain score when compared with the other groups. * indicates significant difference when compared with other Groups (p < 0.05)

The questionnaire was sent to 248 patients, of which 182 (75.4%) returned a full set of data (Fig. 1). The mean age of patients at the time of injury was 53.3 years (SD 17.1), and at the time of questionnaire completion, it was 57.8 years (SD 17.4). Among the patients with pelvic ring injuries, 20 (16.7%) were classified into Group APC, 94 (78.3%) into Group LC, 6 (5.0%) into Group CMVS, and 60 (33.3%) into the Group Control. There were no statistically significant differences in age, rate of peripheral artery disease (PAD), hypertension, hypercholesterinemia, diabetes, or smoking habits between the injured patients and the control group (Table 1). Patients in Group CMVS had significantly lower physical role function scores (62.5, SD 29.6 points) compared to those in Group APC (77.8, SD 27.6 points) or Group LC (79.4, SD 24.7 points, p < 0.001). Additionally, pain scores were worse in Group CMVS (50.0, SD 38.7 points) compared to Group APC (72.5, SD 38.1 points) and Group LC (75.5, SD 26.5 points, p = 0.019) (Fig. 2). Group APC had significantly lower scores on the IIEF-5 (12.2, SD 6.93 points) compared to Group Control (15.97, SD 8.28 points), Group LC (16.23, SD 7.81 points), and Group CMVS (18.67, SD 7.5 points, p = 0.015). A total of 5 patients (25%) suffered an APC Type 1, 7 patients were diagnosed with an APC Type 2 injury (35%) and 8 patients were diagnosed with an APC type 3 (40%) injury. The effect of the type of APC injury did not show a significant impact on reduced IIEF-5 score (p = 0.437). Severe ED was diagnosed in 30% of patients (n = 6) in Group APC, which was significantly higher compared to Groups LC and Control (30% vs. 25.5%, p = 0.049). The rate of severe ED was comparable between Group LC and Group Control (25.5% vs. 23.3%, p = n.s.). None of the patients with an APC type pelvic ring fracture had an IIEF-5 score of 22 or higher, indicating that all these patients suffered from at least a mild form of ED (Table 2). In the univariate analysis, the APC type of pelvic ring injury was associated with a reduction in IIEF-5 score by −3.77 points compared to Group Control (95% CI −7.75 to −0.22, p = 0.006) (Table 3). The multivariate analysis, adjusted for risk factors for ED, revealed that hypertension, diabetes, smoking, and APC-type pelvic ring fracture are independent risk factors for a reduced IIEF-5 score. Specifically, APC type pelvic ring injuries resulted in a reduction of −4.5 points compared to the control group (95% CI −8.26 to −0.74, p = 0.02) (Table 3).

**Table 1:** Demographics of study population

	Pelvic fracture	Group control	p-value
n	120	60	
Age at injury [years], mean (SD)	53.9 (16.7)	52.7 (18.0)	0.641
Age at survey [years], mean (SD)	58.8 (16.4)	55.60 (19.1)	0.24
Type of pelvic ring injury (Y&B), n (%)			
APC	20 (16.7)		
CMVS	6 (5.0)		
LC	94 (78.3)		
ISS [points], mean (SD)	34.86 (13.4)	15.52 (13.1)	< 0.001
Vasculopathy, n (%)	2 (1.7)	6 (10.0)	0.03
PAD, n (%)	0 (0.0)	2 (3.3)	0.209
Hyptertension, n (%)	14 (11.7)	10 (16.7)	0.485
Hypercholesterionemia, n (%)	8 (6.7)	2 (3.3)	0.565
Coronar artery disease, n (%)	10 (8.3)	0 (0.0)	0.05
Diabetes, n (%)	12 (10.0)	2 (3.3)	0.201
Smoking, n (%)	28 (23.3)	10 (17.2)	0.463

*SD* standard deviation, *Y&B* Young and Burgess, *APC* anterior posterior compression, *CMVS* combined mechanism vertical shear, *LC* lateral compression, *ISS* injury severity score, *PAD* peripheral artery disease

**Table 2:** IIEF 5 score distribution according to type of injury

	Group APC	Group LC	Group CMVS	Group Control	p-value
n	20	94	6	60	
IIEF5 [points], mean (SD)	12.20 (6.93)	16.23 (7.81)	18.67 (7.50)	15.97 (8.28)	0.015
Erectile dysfunction, n (%)					0.049
No ED (22–25 points)	0 (0.0)	30 (31.9)	4 (66.7)	22 (36.7)	
Mild ED (17–21 points)	8 (40.0)	24 (25.5)	0 (0.0)	14 (23.3)	
Moderate ED (9–16 points)	6 (30.0)	16 (17.0)	2 (33.3)	10 (16.7)	
Severe ED (0–8 points)	6 (30.0)	24 (25.5)	0 (0.0)	14 (23.3)	

*IIEF 5* international index of erectile function 5, *SD* standard deviation, *APC* anterior posterior compression, *LC* lateral compression, *CMVS* combined mechanism vertical shear, *ED* erectile dysfunction

**Table 3:** Univariate and multivariate analyses for risk factor of reduced IIEF-5 score

	Odds ratio (point reduction IIEF-5)	95%CI	p-value
Univariate analysis			
Group Control	Reference	NA	NA
Group APC	− 3.77	− 7.75 to − 0.22	0.006
Group LC	0.27	− 2.28 to 2.82	0.84
Group CMVS	2.7	− 3.91 to 9.31	0.42
Multivariate analysis			
Group Control	Reference	NA	NA
Group APC	− 4.5	− 8.26 to − 0.74	0.02
Group LC	1.24	− 1.21 to 3.69	0.32
Group CMVS	3.63	− 2.60 to 9.85	0.25
Age at injury [years]	− 0.036	− 0.11 to 0.04	0.35
Vasculopathy	7.01	− 1.43 to 12.59	0.15
Hypertension	− 9.18	− 12.80 to − 5.56	< 0.0001
Coronary artery disease	2.10	− 2.74 to 6.94	0.4
Diabetes	− 5.3	− 9.38 to − 1.22	0.012
Smoking	− 2.61	− 5.25 to 0.04	0.055

*IIEF-5* international index of erectile function 5, *CI* confidence interval, *APC* anterior posterior compression, *LC* lateral compression, *APC* anterior posterior compression, *CMVS* combined mechanism and vertical shear, *NA* not applicable

## Discussion

Pelvic ring fracture represent a challenge in the initial treatment and in the rehabilitation process. After lifesaving surgery, the long-term QoL following pelvic ring injuries depends on the type of injury [18, 19] and the presence of additional injuries [20]. This study aims to investigate the association of pelvic ring fractures and the development of ED. The main results of this study were: CMVS type of pelvic ring injuries are associated with impaired QoL and increased pain. All Patients with APC injuries suffered form of ED.

Numerous studies have investigated the type of injury as a potential risk factor for impaired quality of life [9, 18, 20, 21]. The majority of studies are in accordance with our results that CMVS type of injuries are associated with impaired QoL. The long-term outcome of patients with posterior or combined anterior and posterior injuries is poorer than those with an isolated anterior injury [10]. The severity of the pelvic ring fracture is usually associated increased trauma load and higher trauma energy. The resulted additional injuries and increased instability of the pelvic ring challenge the orthopaedic trauma surgeon. Among the causes of the impaired QoL count the impaired functional outcome. When compared with the population norms, patients who suffered from pelvic ring fractures show impaired functional outcome [8, 22]. The impaired QoL is associated with severity of injury, fracture type, incidence of nerve injuries, or treatment strategy [20].

A recently published Meta-analysis demonstrated that patients with APC fractures results suffer from ED in 29.3% of cases [11]. This rate is higher when compared to the other types of injuries. The presented results are in accordance with the literature and provide some evidence that one in three patients suffers from ED following APC type of pelvic ring fracture. Furthermore, our presented data show that none of the patients with APC injuries had maximum scores in the IIEF-5. It appears that all patients suffer from at least a mild form of ED. The pathophysiology of the association of ED and APC injuries is not yet fully understood. One might argue that most of the trauma energy injures the soft tissue at the anterior portion of the pelvic ring (in APC injuries). This might result in the disruption of the adjacent soft tissue of the anterior pelvic ring [23]. An injury of the urinary tract represent a relevant risk factor for the development of ED [11, 24]. Second, the disruption of vessels and bleeding might impair the blood flow and increase the risk for the development of ED. The internal pudendal artery might be at risk following severe pelvic injuries [25]; the disruption of the vasculature of the pelvis represents a feared complication that might result in devastating hemorrhage [26]. Third, the mechanism of injury might further lead to nerve damages around the pelvis (i.e. pelvic plexus) [24, 27]. Neuro-anatomical variations must be taken into consideration in the assessment and the treatment of pelvic ring fractures [28]. Especially the injuries to the cavernous or pudendal nerve might result in ED [29]. It appears that the combination of soft tissue injuries and injuries to the neuro-vascular structures of the pelvis following APC injuries might be responsible for the development of ED [11]. Injuries to the urethra have been reported to be associated with an increased risk for the development of ED [5, 24].

Numerous studies have investigated medical risk factors for the development of ED: among the most commonly reported risk factors are hypertension, cardiovascular disorders, smoking, obesity, dyslipidemia, diabetes, metabolic syndrome, anxiety and depression [12]. Taken those risk factors into consideration, the present study suggest that APC type of pelvic ring fractures might further represent an independent risk factor for the development of ED.

The presence of an APC-type of pelvic ring injury results in a decrease of −4.5 points on the IIEF-5 scale. This results in a clinically relevant severity of ED [30]. This decrease of IIEF-5 score following APC injuries is comparable to the effect of diabetes (−2.6 points to −3.0 points), heart disease (−2.95 points), or depression (−2.52 points) [30, 31].

## Limitations

This study was designed as a cross sectional study of patients treated at one academic trauma center. The nature of this study design has certain limitations: The cross-sectional study provides data from one random time-point. Further, the presented data are results of self-reported conditions of the patients. Therefore, the results base on the subjective assessment of the patient on their condition. These individual assessments have not been verified by medical professionals. Second, numerous patients did not reply or did not fill out the questionnaire. That might skew the numbers of presented results. However, we are convinced that the reported result the current situation of the patient. The sample size of the present population might be subject to a type 2 error. Especially in the subgroup analysis of APC injuries, the lack of association of severity of injury with the development of ED is at high risk for a false negative result. Finally, the age of the patients (58.8 years) at the questionnaire might represent an independent risk factor for the development of ED. However, the age group is comparable among our study population and the study groups that should not increase the risk of selection bias. Even though that some articles identified age as an independent risk factor for the development of erectile dysfunction, these articles failed to control or correct for age-associated comorbidities such as diabetes, vascular diseases and so on [32]. The questionnaires are well-established tools for the assessment of QoL and ED. Further, the presented results are in accordance with the literature, and therefore might reflect the association of pelvic ring injuries and ED.

## Conclusion

The severity of pelvic ring injuries is closely associated with impaired quality of life, particularly with persistent pain seen in combined mechanism or vertical shear injuries. APC injuries have been identified as an independent risk factor for the development of erectile dysfunction (ED). These findings emphasize the need for early screening and appropriate management of ED in patients with APC-type pelvic ring fractures to improve long-term outcomes.

## Data Availability

No datasets were generated or analysed during the current study.
